# Perception of Health Conditions and Test Availability as Predictors of Adults’ Mental Health during the COVID-19 Pandemic: A Survey Study of Adults in Malaysia

**DOI:** 10.3390/ijerph17155498

**Published:** 2020-07-30

**Authors:** Huiyang Dai, Stephen X. Zhang, Kim Hoe Looi, Rui Su, Jizhen Li

**Affiliations:** 1 School of Economics and Management, Tsinghua University, Beijing 100084, China; daihy1995@163.com; 2 Faculty of Professions, University of Adelaide, Adelaide SA 5000, Australia; 3 School of Economics and Management, Xiamen University Malaysia, Sepang 43900, Selangor, Malaysia; gideon_looi@xmu.edu.my (K.H.L.); ceciliasuuu@outlook.com (R.S.); 4 Research Center for Competitive Dynamics and Innovation Strategy, School of Economics and Management, Tsinghua University, Beijing 100084, China; lijzh@sem.tsinghua.edu.cn

**Keywords:** psychiatric screening, perceived health condition, perceived COVID-19 test availability, risk factors, 2019-nCoV, mental health

## Abstract

Research identifying adults’ mental health during the coronavirus disease 2019 (COVID-19) pandemic relies solely on demographic predictors without examining adults’ health condition as a potential predictor. This study aims to examine individuals’ perception of health conditions and test availability as potential predictors of mental health—insomnia, anxiety, depression, and distress—during the COVID-19 pandemic. An online survey of 669 adults in Malaysia was conducted during 2–8 May 2020, six weeks after the Movement Control Order (MCO) was issued. We found adults’ perception of health conditions had curvilinear relationships (horizontally reversed J-shaped) with insomnia, anxiety, depression, and distress. Perceived test availability for COVID-19 also had curvilinear relationships (horizontally reversed J-shaped) with anxiety and depression. Younger adults reported worse mental health, but people from various religions and ethnic groups did not differ significantly in reported mental health. The results indicated that adults with worse health conditions had more mental health problems, and the worse degree deepened for unhealthy people. Perceived test availability negatively predicted anxiety and depression, especially for adults perceiving COVID-19 test unavailability. The significant predictions of perceived health condition and perceived COVID-19 test availability suggest a new direction for the literature to identify the psychiatric risk factors directly from health-related variables during a pandemic.

## 1. Introduction

In May 2020, the United Nations Secretary-General issued a message that the coronavirus disease 2019 (COVID-19) pandemic had resulted in massive mental suffering and called for actions [1]. Research is critically needed on mental health of patients, healthcare workers, and the general population during the COVID-19 pandemic. Distress, anxiety, depression, and insomnia are relevant mental health disorders due to their prevalence during severe acute respiratory syndrome (SARS) and COVID-19 outbreaks [2,3,4,5]. A number of studies have predicted mental health by means of demographic variables [2,3,6], but little research has predicted mental health during the COVID-19 pandemic based on adults’ health-related variables. This study aims to examine individuals’ perception of health conditions and of test availability as predictors of insomnia, anxiety, depression, and distress during the COVID-19 pandemic. This study is the first, to the best of our knowledge, to identify such health-related predictors, specifically adults’ perceived health conditions and perceived availability of COVID-19 testing.

We focused on health-related variables as predictors of mental health for several reasons. First, comorbidities, such as diabetes and heart failure, were found to link to more severe fatality rates in the ongoing COVID-19 pandemic [7,8,9]. Second, the Short-Form (SF) Health Survey was found to be negatively associated with mental disorders [10], presenting a case to test the association under the ongoing COVID-19 pandemic. Third, good health conditions can lower individuals’ chance of COVID-19 infection [11,12]. Fourth, health conditions can likely be useful to screen mental health because healthcare workers (e.g., general practitioners) may already have some knowledge of the health status of people under their care. Lastly, due to limited testing capacity in many countries, individuals still have heterogeneous access to COVID-19 testing, and individuals who have poorer access to COVID-19 testing may be more concerned or anxious about the COVID-19 pandemic. Granted, there might be differences between the perceived and actual test availability. Still, most people lack data on the actual test availability and must rely on their perceptions, introducing additional individual-level heterogeneity. Hence, perceived test availability for COVID-19 presents a potentially unique predictor of mental health during the COVID-19 pandemic.

We tested the predictors empirically in Malaysia, an upper middle income nation in South East Asia. In 2020, the Malaysian Ministry of Health has received RM 30.6 billion (10.2%) out of RM 299 billion national budget. Like many other countries, Malaysia is susceptible to public health crises, such as the Nipah virus in 1999, Severe Acute Respiratory Syndrome in 2003, and Middle East Respiratory Syndrome Coronavirus in 2018. The first case of COVID-19 in Malaysia was confirmed on 4 February 2020. On 18 March 2020, Malaysia implemented a Movement Control Order (MCO) to ban citizens from non-essential travel and mass gatherings. Prior to the outbreak of COVID-19, mental problems were an already prevalent health issue in Malaysia [13].

## 2. Materials and Methods

The data for this study were collected by an online survey from May 2 to 8, 2020, six weeks after the implementation of the MCO in Malaysia. On 8 May 2020, there were a total of 6535 confirmed cases of COVID-19 and 108 deaths [14]. We applied a two-stage stratified sampling. In the first stage, we did stratified sampling based on regions, specifically the principal administrative divisions in Malaysia of 13 states (Negeri) and 3 federal territories (Wilayah Persekutuan). In the second stage, we did cluster-sampling based on the ethnicity, gender, and age groups [15] of the Malaysian population [16]. To minimize response and measurement bias, we followed the standard survey approaches [17], i.e., no social pressure to influence responses, no questions that provoke defensiveness or threaten esteem, no payoff or cost for particular responses. Multi-item questions were used to ensure no priming, and there was no overlapping among questions for different constructs [18]. Participation in this survey was voluntary, and participants could opt out at any time. Moreover, participants were assured anonymity and confidentiality of their responses. The survey was granted ethical approval by Tsinghua University (20200322). The online survey was issued in Malay, Mandarin, and English, the three major languages used in Malaysia. Given that the survey was entirely voluntary, and the introduction provided the estimated minutes to complete the survey as well as consent form, 89.7% of the adults (669 out 746) who consented to participate finished the survey. All of the 669 participants were found to be above 18 years old and, hence, fulfilled the eligibility of the criterion of adulthood in Malaysia to be included in this study.

The participants reported their demographic characteristics, including gender, age, education level, number of children under 18 years old in the household, religion, and ethnic group. We assessed health condition using the global health measure SF-1 [19] with a five-point scale from 1 to 5 (poor, fair, good, very good, excellent). To capture perceived availability of testing for COVID-19 in Malaysia, we asked participants to rate the statement “I can get a test for COVID-19 rapidly if I need it” from 1 to 7 (strongly disagree, disagree, somewhat disagree, neither agree nor disagree, somewhat agree, agree, strongly agree). 

We used four dimensions for mental health: insomnia, anxiety, depression, and distress. They were all measured with commonly used established scales in literature. To examine the reliability of each construct, Cronbach’s alpha was computed [20].

### 2.1. Insomnia

Adults’ insomnia was measured with the five-item Athens Insomnia Scale (AIS-5) [21], including “I have trouble falling asleep” and “I feel tired and worn-out after my usual amount of sleep”. The items were scored from 1 (to a very small extent) to 5 (to a very large extent). The Cronbach’s alpha was 0.82. The average score constructed with Cronbach’s alpha was used to evaluate insomnia. 

### 2.2. Anxiety

Adults’ anxiety was measured by the seven-item generalized anxiety disorder (GAD-7) [22] scale. The seven items were scored from 0 (not at all) to 3 (nearly every day). The Cronbach’s alpha was 0.92. The total score of seven items was used to evaluate anxiety.

### 2.3. Depression

Adults’ depression was measured with the nine-item Patient Health Questionnaire depression module (PHQ-9) [23], with items scored from 0 (not at all) to 3 (nearly every day). The Cronbach’s alpha was 0.90. The total score of nine items was used to evaluate depression.

### 2.4. Distress

Adults’ psychological distress was measured with the six-item K6 screening scale [24], with items scored from 1 (all of the time) to 5 (none of the time). The Cronbach’s alpha was 0.95. The total score of six items was used to evaluate depression.

We analyzed the data using STATA software version 16.0 (StataCorp LLC, College Station, Texas, USA). We used multivariable least-squares regression analysis to predict the risk factors for the mental health disorders of Malaysian adults at a significance level of 0.05. The associations between risk factors and outcomes were adjusted for confounders, following other psychiatric studies under COVID-19 [25].

## 3. Results

### 3.1. Descriptive Findings

Overall, 669 adults from all the states and federal territories of Malaysia participated in this survey. (Data will be available on request for the readers.) Table 1 shows the descriptive statistics of the sample. Participation by both genders was almost equal. The youngest participant was 21 years old and the oldest was 71 years old. Malaysia is a diverse country in terms of ethnicity and religion. Religion and ethnicity are reported in Table 1. Overall, our sample captured all the major ethnic and religious groups in Malaysia, but the sample is not taken as representative.

According to Table 1, the mean scores of insomnia (AIS-5), anxiety (GAD-7), depression (PHQ-9), and distress (K6) were 1.76 (standard deviation (SD) = 0.84), 4.36 (SD = 4.89), 4.49 (SD = 5.03), and 5.10 (SD = 5.73), respectively.

### 3.2. Predictors of Insomnia, Anxiety, Depression, and Distress

Table 2 presents the results of the regression models. The quadratic terms of health condition (health condition—square in Table 2) were significantly positive across the regressions for all four dimensions of mental health: insomnia, anxiety, depression, and distress. This demonstrates curvilinear relationships between health condition and mental health dimensions. The margin analysis of the slope of insomnia by health condition was −0.71 (*p* < 0.001) at “poor”, −0.51 (*p* < 0.001) at “fair”, −0.31 (*p* < 0.001) at “good”, −0.10 (*p* = 0.017) at “very good”, and 0.10 (*p* = 0.328) at “excellent” health condition, showing a horizontally reversed J-shaped curve across the scoring range of health condition. Similar curvilinear relationships were also observed for anxiety, depression, and distress. The slope of anxiety was −5.34 (*p* < 0.001) at “poor”, −3.76 (*p* < 0.001) at “fair”, −2.18 (*p* < 0.001) at “good”, −0.60 (*p* = 0.019) at “very good”, and 0.98 (*p* = 0.114) at “excellent” health condition. The slope of depression was −5.04 (*p* < 0.001) at “poor”, −3.55 (*p* < 0.001) at “fair”, −2.06 (*p* < 0.001) at “good”, −0.57 (*p* = 0.024) at “very good”, and 0.91 (*p* = 0.104) at “excellent” health condition. The slope of distress was −3.11 (*p* = 0.006) at “poor”, −2.21 (*p* = 0.002) at “fair”, −1.32 (*p* < 0.001) at “good”, −0.43 (*p* = 0.188) at “very good”, and 0.46 (*p* = 0.511) at “excellent” health condition.

The quadratic term of test availability (test availability—square in Table 2) was positively associated with anxiety and depression. The margin analysis of the slope of anxiety by test availability was −1.49 (*p* < 0.004) at “strongly disagree”, −1.15 (*p* = 0.002) at “disagree”, −0.81 (*p* < 0.001) at “somewhat disagree”, −0.48 (*p* < 0.001) at “neither disagree nor agree”, −0.14 (*p* = 0.311) at “somewhat agree”, 0.19 (*p* = 0.423) at “agree”, and 0.53 (*p* = 0.152) at “strongly agree”, showing a horizontally reversed J-shaped curve across the scoring range of test availability. The slope of depression by test availability demonstrated a similar pattern at the 7-point anchor: −1.41 (*p* = 0.005) at “strongly disagree”, −1.10 (*p* = 0.003) at “disagree”, −0.79 (*p* < 0.001) at “somewhat disagree”, −0.48 (*p* < 0.001) at “neither disagree nor agree”, −0.17 (= 0.263) at “somewhat agree”, 0.16 (*p* = 0. 562) at “agree”, and 0.46 (*p* = 0.227) at “strongly agree”.

**Figure 1:**
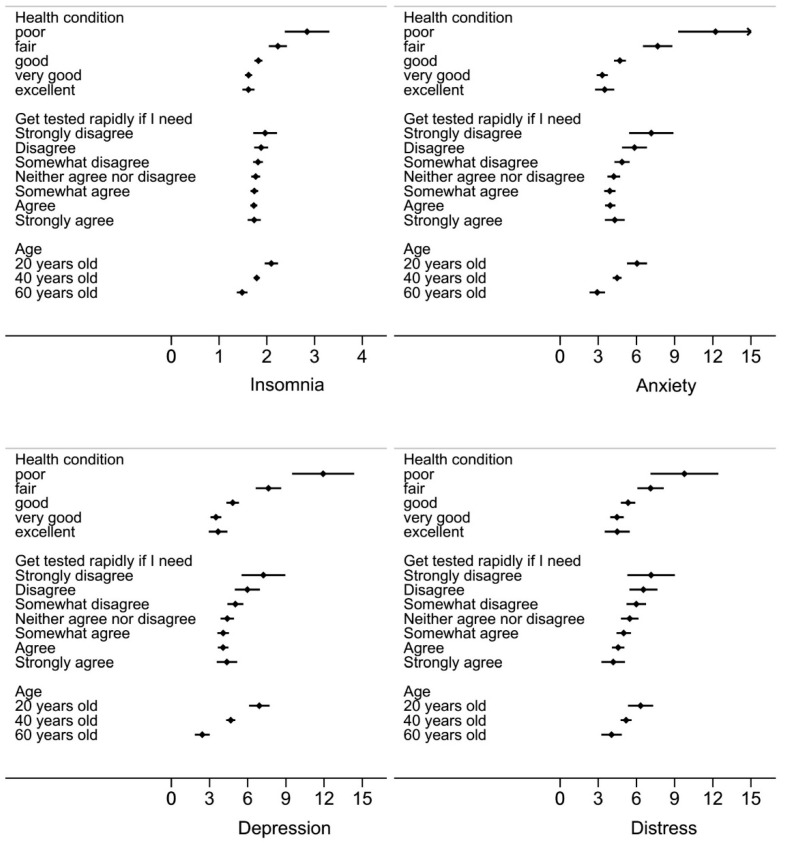
Predicted value and 95% confidence intervals (CIs) of insomnia, anxiety, depression, and distress scores by health condition, COVID-19 test availability, and age.

In addition, age negatively predicted insomnia, anxiety, depression, and distress as shown in Table 2. Insomnia, anxiety, depression, and distress did not vary significantly by religion and ethnicity. The predicted values of insomnia, anxiety, depression, and distress by health conditions, test availability, and age are shown in Figure 1.

## 4. Discussion

This study identified adults’ perceived health conditions, perceived test availability, and age as the predictors of their insomnia, anxiety, depression, and distress during the COVID-19 pandemic. The average levels of insomnia, anxiety, depression, and distress of this study in Malaysia are different from adults in other countries under the COVID-19 pandemic (Table 1). The mean scores of depression and anxiety in Malaysia were significantly lower than those in a sample of 300 adults collected on 31 January to 7 February 2020 in China [21] of 8.3 and 7.7, and also lower than scores in a sample of 1009 adults on 10–20 April 2020 in Austria [22] of 6.20 and 5.85. The average level of distress in a sample of 369 working adults (mean = 8.46) in China [23] was significantly higher than in our sample. The proportion of insomnia disorder in our sample (38.9%, *n* = 669) was approximate to that in a sample collected during 10–13 April 2020 in Greece (37.6%, *n* = 2427) during the COVID-19 pandemic [24].

Consistent with past studies [6,26], age was found to be a predictor of mental health problems for the general population in Malaysia. This is also consistent with studies of healthcare workers, where older healthcare workers were less likely to have mental health problems [27,28]. However, other predictors found in the literature, such as education [25] and gender [29], failed to predict mental health among adults in Malaysia, similar to a study in the UK [6]. Our results suggest that future research should identify the effect of education and gender across more countries, and future meta-analysis should identify specific contingent factors. Religion and ethnic groups did not predict Malaysian adults’ mental health, consistent with another Malaysian study [30] which found that ethnicity was not correlated with mental health disorders. In line with previous research, our findings highlight the need to identify specific predictors of mental health under various contexts of the COVID-19 pandemic [27].

More importantly, this study uncovers two unique risk factors for mental health. The first risk factor is existing health condition. Previous research found positive associations between physical health condition and mental disorders [7,10], yet we found that individuals’ health conditions had significant curvilinear relationships with their insomnia, anxiety, depression, and distress. The results indicated that adults with worse health conditions had worse mental health, and this association was more negative for those at the lower end of the health spectrum. Our findings suggest individuals’ perceived health conditions can be a useful screener of insomnia, anxiety, depression, and distress during the COVID-19 pandemic.

The second risk factor is perceived test availability for COVID-19, which had curvilinear relationships with anxiety and depression. Perceived test unavailability predicted worse anxiety and depression, especially for people who disagreed that they could get tested for COVID-19 when needed. There was also no significant difference in mental health among people who “somewhat agreed”, “agreed”, and “strongly agreed” that they could get a COVID-19 test. Most people lack data on the actual test availability, and they must rely on their perceptions. We believe perceived test availability could be a predictor of mental health, especially among adults who lack access to COVID-19 tests.

Our findings suggest that healthcare service providers could use adults’ perceived health conditions and perceived COVID-19 test availability to identify mentally vulnerable adults. The curvilinear relationships highlight the need to pay more attention to adults with perceived poor health conditions and adults who believe they lack access to a COVID-19 test. Healthcare service providers such as hospitals may be able to use the prior health records of their patients and COVID-19 testing coverage to help identify those who need more mental health assistance.

The study has several limitations. Firstly, because we aimed to identify predictors for healthcare service providers in developing screening for mental disorders, cross-sectional data were used and should not be taken as evidence of causality. Secondly, we used SF-1, a brief one-item measure of general health condition, and future research may use the lengthier form of SF-12 or SF-36. Thirdly, instead of perceived health conditions, future research may explore specific medical issues, such as heart disease, diabetes, or cancer, as predictors of mental health. Fourthly, we measured adults’ perceived test availability for COVID-19 because we were interested in their mental health, and future research may use alternative indicators of COVID-19 test availability. Finally, because we sent out questionnaires online, only adults who have access to the internet in Malaysia could participate in the survey. The internet penetration rate in Malaysia (86% in 2018) [31] might introduce another source of limitation on the generalizability.

## 5. Conclusions

This study identified two unique predictors of mental health in individuals’ perceived health conditions and perceived COVID-19 test availability. Perceived health condition predicted insomnia, anxiety, depression, and distress, and perceived test availability predicted anxiety and depression. Unlike the demographic predictors identified in prior research, this study suggests two new risk factors to predict mental health problems. Moreover, these predictors carried quadratic associations with various mental health dimensions, implying a need to focus on curvilinear predictors of mental health.

## Figures and Tables

**Table 1:** Descriptive statistics of Malaysian participants.

Variables	Description *n* (%)	Insomnia	Anxiety	Depression	Distress
Mean (SD ^1^)	669 (100%)	1.76 (0.84)	4.36 (4.89)	4.49 (5.03)	5.10 (5.73)
Min		1	0	0	0
Max		5	21	27	24
		**Mean (SD)**			
**Gender**					
Male	324 (48.43%)	1.69 (0.76)	4.04 (4.68)	4.02 (4.54)	5.25 (6.00)
Female	345 (51.57%)	1.84 (0.91)	4.66 (5.06)	4.93 (5.43)	4.96 (5.48)
**Age (years old)**					
20–29	100 (14.95%)	1.89 (0.79)	5.29 (5.00)	5.95 (4.72)	5.78 (5.83)
30–39	197 (29.45%)	1.99 (0.91)	5.12 (5.10)	5.64 (5.67)	5.54 (5.89)
40–49	192 (28.70%)	1.65 (0.81)	4.37 (4.78)	4.16 (4.85)	5.02 (5.73)
50–59	148 (22.12%)	1.58 (0.76)	3.07 (4.32)	3.03 (4.19)	4.62 (5.77)
60–71	32 (4.78%)	1.52 (0.74)	2.69 (4.80)	1.56 (2.84)	2.88 (3.27)
**Education level**					
Secondary school	49 (7.32%)	1.71 (0.72)	4.63 (4.72)	4.08 (5.24)	6.08 (5.77)
College or university	406 (60.69%)	1.80 (0.86)	4.42 (4.81)	4.69 (4.97)	5.17 (5.71)
Graduate school	214 (31.99%)	1.70 (0.83)	4.18 (5.08)	4.20 (5.11)	4.73 (5.75)
**Number of children in household**					
0	322 (48.13%)	1.78 (0.85)	4.30 (4.72)	4.77 (5.02)	4.79 (5.10)
1	114 (17.04%)	1.69 (0.82)	4.31 (5.18)	4.18 (5.02)	5.44 (6.63)
2	101 (15.10%)	1.68 (0.73)	4.21 (4.28)	3.89 (4.20)	5.61 (5.63)
≥3	132 (19.73%)	1.77 (0.92)	4.48 (5.47)	4.25 (5.63)	5.36 (6.41)
**Religion**					
Islam	352 (52.62%)	1.80 (0.88)	4.53 (5.06)	4.64 (5.24)	5.12 (5.80)
Buddhism	112 (16.74%)	1.66 (0.74)	4.38 (4.62)	4.39 (4.62)	4.97 (5.37)
Hinduism	24 (3.59%)	1.93 (0.83)	5.00 (6.47)	4.67 (5.47)	6.17 (6.03)
Traditional Chinese religion	26 (3.89%)	1.63 (0.78)	3.62 (3.61)	4.31 (4.92)	4.58 (5.93)
Sikhism	6 (0.90%)	2.08 (1.06)	9.83 (7.91)	7.17 (5.38)	5.83 (4.12)
Christianity/Catholic	124 (18.54%)	1.73 (0.82)	3.44 (3.76)	3.74 (4.42)	4.99 (5.98)
Others	3 (0.45%)	1.33 (0.58)	0.67 (1.15)	0.67 (1.15)	0.67 (1.15)
None	22 (3.29%)	1.91 (0.94)	6.05 (6.21)	6.68 (6.19)	5.82 (5.42)
**Ethnic group**					
Malay	328 (49.03%)	1.78 (0.87)	4.41 (5.02)	4.59 (5.23)	5.07 (5.80)
Chinese	221 (33.03%)	1.65 (0.76)	3.91 (4.32)	4.18 (4.58)	4.77 (5.44)
Indian	36 (5.38%)	1.94 (0.87)	5.86 (6.70)	5.00 (5.17)	7.22 (6.61)
Bumiputra of Sabah and Sarawak	75 (11.21%)	1.93 (0.90)	4.84 (4.82)	4.56 (5.18)	5.09 (5.47)
Others	9 (1.35%)	1.78 (0.74)	3.67 (4.50)	5.67 (6.98)	5.44 (8.03)
**COVID-19 test availability**					
strongly disagree	33 (4.93%)	1.98 (1.02)	7.97 (7.65)	8.24 (7.32)	7.55 (6.84)
Disagree	34 (5.08%)	1.92 (1.01)	5.26 (5.12)	4.79 (5.12)	6.06 (6.83)
Somewhat disagree	42 (6.28%)	1.79 (0.69)	3.98 (4.26)	4.05 (4.25)	6.98 (7.43)
Neither agree nor disagree	150 (22.42%)	1.80 (0.89)	4.49 (4.40)	5.00 (5.50)	5.33 (5.65)
Somewhat agree	94 (14.05%)	1.73 (0.71)	3.77 (4.04)	4.02 (3.79)	4.85 (5.69)
Agree	227 (33.93%)	1.63 (0.76)	3.69 (4.42)	3.54 (4.16)	4.34 (4.86)
strongly agree	89 (13.30%)	1.93 (0.99)	4.98 (5.85)	5.25 (5.90)	4.72 (5.83)
**Health condition**					
Poor	7 (1.05%)	3.00 (1.41)	13.29 (9.84)	13.57 (7.46)	9.71 (7.54)
Fair	74 (11.06%)	2.15 (1.00)	7.41 (5.78)	7.09 (5.80)	6.85 (5.52)
Good	209 (31.24%)	1.80 (0.82)	4.46 (4.63)	4.63 (5.09)	4.89 (5.15)
Very good	250 (37.37%)	1.63 (0.74)	3.47 (3.91)	3.65 (4.12)	4.93 (5.98)
Excellent	129 (19.28%)	1.66 (0.80)	3.68 (4.96)	3.91 (5.01)	4.51 (5.94)

^1^ standard deviation.

**Table 2:** Predictors of adults’ insomnia, anxiety, depression and distress by regression analyses (*n* = 669).

Variables	Insomnia		Anxiety		Depression		Distress	
	*β* (95% CI ^1^)	*p*-Value	*β* (95% CI)	*p*-Value	*β* (95% CI)	*p*-Value	*β* (95% CI)	*p*-Value
**Health condition–square**	0.10 (0.03 to 0.17)	0.003	0.79 (0.36 to 1.22)	0.000	0.74 (0.38 to 1.11)	0.000	0.45 (0.01 to 0.88)	0.045
**Health condition**	−0.91 (−1.41 to −0.42)	0.000	−6.91 (−10.06 to −3.77)	0.000	−6.53 (−9.18 to −3.88)	0.000	−4.00 (−7.08 to −0.91)	0.011
**Test availability–square**	0.01 (−0.01 to 0.03)	0.404	0.17 (0.03 to 0.31)	0.017	0.16 (0.02 to 0.29)	0.027	0.02 (−0.14 to 0.18)	0.790
**Test availability**	−0.11 (−0.30 to 0.08)	0.253	−1.82 (−3.10 to −0.55)	0.005	−1.73 (−2.99 to −0.46)	0.007	−0.67 (−2.08 to 0.75)	0.355
**Gender (Female)**	0.08 (−0.05 to 0.21)	0.214	0.22 (−0.48 to 0.93)	0.535	0.49 (−0.24 to 1.22)	0.190	−0.54 (−1.40 to 0.32)	0.217
**Age**	−0.02 (−0.02 to −0.01)	0.000	−0.08 (−0.11 to −0.05)	0.000	−0.11 (−0.14 to −0.08)	0.000	−0.06 (−0.10 to −0.02)	0.005
**Education level**	−0.05 (0.36 to −0.15)	0.056	−0.25 (−0.86 to 0.36)	0.414	−0.10 (−0.74 to 0.55)	0.770	−0.64 (−1.40 to 0.12)	0.100
**Number of children**	0.02 (−0.02 to 0.07)	0.331	0.16 (−0.09 to 0.41)	0.209	0.02 (−0.27 to 0.30)	0.912	0.25 (−0.07 to 0.56)	0.131
**Religion**								
Islam	Reference group		Reference group		Reference group		Reference group	
Buddhism	−0.12 (−0.54 to 0.31)	0.593	−0.02 (−2.39 to 2.35)	0.986	0.61 (−1.75 to 2.96)	0.613	0.15 (−2.66 to 2.97)	0.916
Hinduism	0.08 (−0.62 to 0.79)	0.814	−0.02 (−4.10 to 4.07)	0.994	1.13 (−2.77 to 5.04)	0.569	−3.34 (−10.00 to 3.32)	0.325
Traditional Chinese religion	−0.15 (−0.64 to 0.35)	0.561	−0.80 (−3.29 to 1.69)	0.529	0.71 (−2.09 to 3.51)	0.618	−0.37 (−3.89 to 3.16)	0.837
Sikhism	0.11 (−0.8 to 1.03)	0.807	3.78 (−2.57 to 10.13)	0.243	3.31 (−1.23 to 7.84)	0.153	−4.90 (−11.86 to 2.07)	0.168
Christianity/Catholic	−0.12 (−0.51 to 0.27)	0.546	−1.56 (−3.71 to 0.58)	0.154	−0.22 (−2.39 to 1.95)	0.840	−0.16 (−2.65 to 2.33)	0.900
Others	−0.77 (−1.89 to 0.34)	0.175	−6.10 (−11.52 to −0.69)	0.027	−5.64 (−10.54 to −0.74)	0.024	−6.22 (−10.94 to −1.50)	0.010
None	0.12 (−0.40 to 0.64)	0.642	1.40 (−1.90 to 4.69)	0.406	2.70 (−0.46 to 5.85)	0.094	0.42 (−2.98 to 3.83)	0.807
**Ethnic group**								
Malay	Reference group		Reference group		Reference group		Reference group	
Chinese	−0.03 (−0.45 to 0.39)	0.873	−0.03 (−2.30 to 2.23)	0.977	−1.08 (−3.33 to 1.16)	0.343	−0.31 (−2.98 to 2.37)	0.823
Indian	0.17 (−0.49 to 0.82)	0.622	1.24 (−2.30 to 4.78)	0.491	−0.78 (−4.17 to 2.61)	0.651	5.30 (−1.23 to 11.84)	0.111
Bumiputra of Sabah and Sarawak	0.19 (−0.19 to 0.58)	0.329	1.25 (−0.91 to 3.41)	0.256	−0.38 (−2.52 to 1.77)	0.731	0.07 (−2.22 to 2.36)	0.951
Others	0.24 (−0.32 to 0.80)	0.405	0.80 (−2.59 to 4.20)	0.642	1.59 (−2.34 to 5.52)	0.427	2.16 (−4.09 to 8.42)	0.497

^1^—confidence interval.
